# In tribute to Bernard Witholt

**DOI:** 10.1111/1751-7915.12293_3

**Published:** 2015-06-15

**Authors:** Willem M de Vos

**Affiliations:** Laboratory of Microbiology, Wageningen UniversityDreijenplein 10, 6703 HB, Wageningen, The Netherlands

It was in the mid-seventies that I met Bernard for the first time – he had just arrived in Groningen as a new professor with fresh ideas. I remember a meeting where I participated as a young student with some older Dutch professors who were, and I say this politely, not amused about the research subjects that Bernard discussed in his inspiring style. It was about *Pseudomonas* growing in a two-phase reactor and its application potential as well its potential for collaborations. This did not go well with the audience as Bernard provided his visionary views for microbial biotechnology in the sense that he wanted disciplines to collaborate and not remain in splendid isolation in their ivory towers. I remember being captivated by his ideas and did not agree with the displayed skepticism and accusations of modernisms of others. Fortunately, the concepts did not disappear from the agenda and in mid-eighties, the Netherlands Society for Biotechnology was founded by Bernard and other inspired colleagues. This provided the basis for further concentration of multidisciplinary activities and their spin-outs. When I became chairman of the Society about 20 years later, Bernard was at ETH Zurich and I invited him to deliver the first key note speech at the bi-annual Netherlands Biotechnology Congress. He provided his views on the innovation that originates from small research groups under the title ‘*Is little science still viable*’. Once again, he inspired many of us – as he did all his life.

